# Impact of Proton Irradiation on Medium Density Polyethylene/Carbon Nanocomposites for Space Shielding Applications

**DOI:** 10.3390/nano13071288

**Published:** 2023-04-06

**Authors:** Federica Zaccardi, Elisa Toto, Shreya Rastogi, Valeria La Saponara, Maria Gabriella Santonicola, Susanna Laurenzi

**Affiliations:** 1 Department of Astronautical Electrical and Energy Engineering, Sapienza University of Rome, Via Salaria 851-881, 00138 Rome, Italy; 2 Department of Chemical Engineering Materials Environment, Sapienza University of Rome, Via del Castro Laurenziano 7, 00161 Rome, Italy; elisa.toto@uniroma1.it (E.T.); mariagabriella.santonicola@uniroma1.it (M.G.S.); 3 Department of Mechanical and Aerospace Engineering, University of California Davis, One Shields Ave, Davis, CA 95616, USAvlasaponara@ucdavis.edu (V.L.S.)

**Keywords:** radiation shielding ability, polyethylene nanocomposites, space environmental degradation, proton irradiation, surface analysis

## Abstract

The development of novel materials with improved radiation shielding capability is a fundamental step towards the optimization of passive radiation countermeasures. Polyethylene (PE) nanocomposites filled with carbon nanotubes (CNT) or graphene nanoplatelets (GNP) can be a good compromise for maintaining the radiation shielding properties of the hydrogen-rich polymer while endowing the material with multifunctional properties. In this work, nanocomposite materials based on medium-density polyethylene (MDPE) loaded with different amounts of multi-walled carbon nanotubes (MWCNT), GNPs, and hybrid MWCNT/GNP nanofillers were fabricated, and their properties were examined before and after proton exposure. The effects of irradiation were evaluated in terms of modifications in the chemical and physical structure, wettability, and surface morphology of the nanocomposites. The aim of this work was to define and compare the MDPE-based nanocomposite behavior under proton irradiation in order to establish the best system for applications as space shielding materials.

## 1. Introduction

Polyethylene (PE) is a widely researched material that finds application in different fields due to its low weight, low cost, and easy processability. Its high biocompatibility, good mechanical properties, and chemical resistance have made PE the material of choice for the commercial production of orthopedic prostheses and packaging [1,2]. In the space sector, PE and PE-based composites are used as a barrier against the hazardous space radiation environment [3,4]. In fact, it is widely recognized that materials composed of low atomic number atoms offer protection against radiation [5]. PE is composed of the ethylene monomer –[CH_2_–CH_2_]– with high content of H atoms and is, therefore, the solid material with the most efficient radiation shielding properties [6]. However, PE is a dielectric polymer and does not possess enough strength and thermal stability to be considered as a structural material [5]. For this reason, carbon nanoparticles are widely investigated for the fabrication of novel PE nanocomposite materials with potentially enhanced mechanical and functional properties: high electrical conductivity for static charge dissipation, high thermal conductivity, radiation hardness, and mechanical integrity [5,6,7,8,9,10]. Many investigations have been reported on the multifunctional properties of nanocomposites based on PE loaded with graphene nanoplatelets (GNP) or carbon nanotubes (CNT), where the PE matrix takes advantage of the exceptionally high strength-to-weight ratio and of the high thermal and electrical conductivities of carbon nanoparticles [11,12,13]. Moreover, several studies report on the effects of different types of irradiation on PE and carbon-filled PE composites, including cross-linking, chain scissions, formation of free radicals, and release of hydrogen [14,15,16,17]. Nevertheless, if the effects of radiation on PE are well-known, studies on the response to radiation of CNTs and GNPs have been limited. However, several studies report applications of graphene/polymer nanocomposites for radiation sensing [18,19,20,21,22], exploiting the high electrical conductivity of the carbon filler. The numerical and experimental studies conducted so far indicate that CNTs are radiation-tolerant nanoparticles [23,24]. The current literature suggests possible beneficial effects of radiation that lead to the formation of inter-tube covalent bonds and of stable cross-links between neighboring CNTs within bundles, thus eliminating the sliding between the nanotubes and increasing their mechanical properties [25,26]. Regarding CNTs nanocomposites, a beneficial effect of the nanoparticles has been observed by many authors [27,28,29,30]. The experimental results on the effects of different types of radiation showed that the addition of CNTs can improve the shielding ability of the polymeric matrices [27,28,29,30]. Wilkins et al. [31] investigated, using Raman spectroscopy, the effects of different simulated space radiation environments on PE loaded with raw (non-functionalized) and functionalized single-walled carbon nanotubes (SWCNT). Results showed that SWCNTs are highly radiation tolerant to space radiation environments, but further studies with additional experimental techniques must be implemented to understand the mechanisms of material–radiation interactions. Contrary to CNTs, studies on the effects of different radiation sources on GNPs report the formation of topological defects such as the ejection of multiple atoms and consequent formation of vacancies [32,33]. However, improvement of radiation resistance, less crystal defects and self-healing mechanism of radiation-induced defects are also observed in GNP nanocomposites by many authors [34,35,36,37,38]. Overall, only few experiments with preliminary results have been conducted to investigate the radioprotectant properties of carbon-based polymer nanocomposites, and the impact of radiation on PE nanocomposites is still unknown.

In this work, nanocomposite materials based on medium-density polyethylene (MDPE) loaded with different amounts of multi-walled carbon nanotubes (MWCNT), GNPs, and hybrid MWCNT/GNP nanofillers are fabricated by a molding technique. The nanocomposites are investigated in terms of chemical structure, thermal behavior, wettability, and morphology, before and after proton irradiation. Results are useful to guide the application of these materials in space environments characterized by high levels of ionizing radiation.

## 2. Materials and Methods

Medium-density polyethylene (MDPE) in the form of powder was supplied by Sigma Aldrich (product code 332119, density 0.94 g/cm^3^) and used as received. Exfoliated graphene nanoplatelets of grade C750 (thickness ~2 nm, average diameter <2 μm, specific surface area ~750 m^2^g^−1^) were purchased from XG Sciences (Lansing, MI, USA) and used as received. Non-functionalized MWCNTs (NC7000 series, outer diameter ~9.5 nm, average length ~1.5 μm, specific surface area ~250–300 m^2^g^−1^) were purchased from Nanocyl S.A. (Sambreville, Belgium) and used as received. The GNP, MWCNT, and MDPE powders were mechanically mixed for 15 min. The mixed powders were used to create samples (1 cm × 1 cm × 0.15 cm) of MDPE/GNP at 2.5 wt%, 5 wt%, 10 wt%, and 15 wt%, of MDPE/MWCNT at 2.5 wt% and 5 wt%, and of MDPE loaded with 20 wt% of GNP/MWCNT hybrid filler in the ratio 3:1 (hereafter referred to as GNP_3_MWCNT_1_) by melting in oven at 125 °C while degassing. 

**Figure 1:**
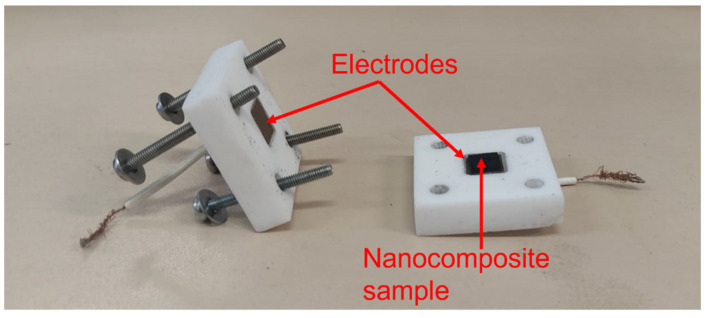
Set-up for electrical measurements: specimen (1 cm × 10 cm × 0.15 mm) placed in the custom-made Teflon cell and contacted by means of flat copper electrodes on the top and bottom surface.

The volumetric electrical conductivity of the specimens was determined by electrical impedance spectroscopy (EIS) using a Reference 600 Potentiostat/Galvanostat/ZRA instrument (Gamry Instruments, Warminster, PA, USA) in the frequency range 10 Hz–1 MHz. The specimens were placed in a custom-made Teflon cell and contacted by means of flat copper electrodes on the top and bottom sample surfaces (Figure 1). The cell was closed in a Faraday cage (Gamry Instruments) to shield the measurements from undesired noise. Impedance data were fitted to an equivalent circuit model using the Gamry Echem Analyst software package. The electrical resistance values (R_s_) obtained from the fitting procedure were converted to conductivities (σ_v_ = 1/ρ_v_), where ρ_v_ is the volumetric resistivity evaluated according to the ASTM D257-07.

**Figure 2:**
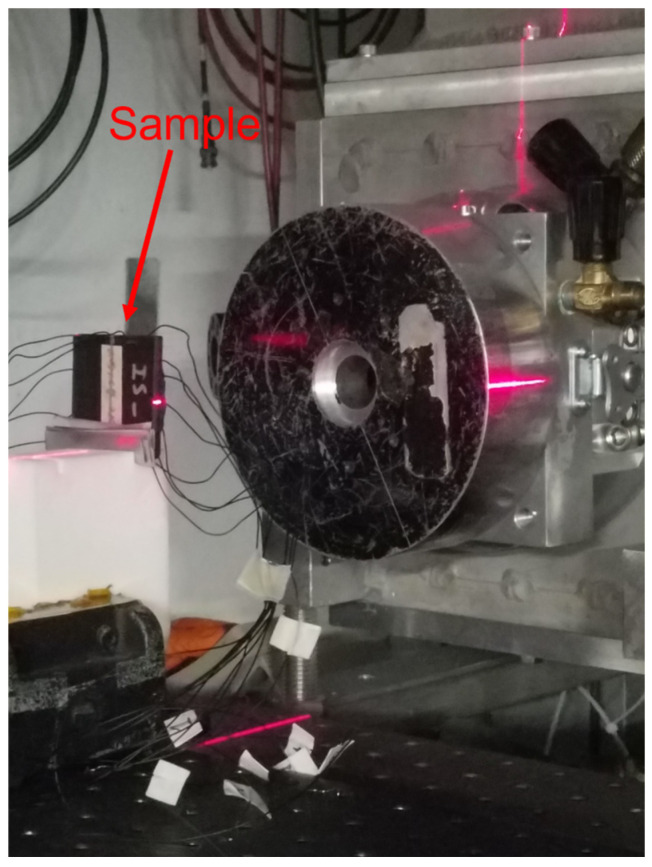
Sample of MDPE/GNP 5 wt% in proton irradiation line.

Based on the electrical measurements, samples (5 cm × 5 cm × 0.5 cm) of MDPE/GNP at 5 wt%, 10 wt%, and 15 wt%, of MDPE/MWCNT at 5 wt%, and of MDPE/GNP_3_MWCNT_1_ were fabricated and subjected for an average time of 294 s to proton irradiation, at an energy of 64 MeV, current 1 nA, for a total dose of 50 Gy. The US Center for Diseases Control and Prevention on Acute Radiation Syndrome reports 50 Gy as the dose causing the fatal collapse of human cardiovascular and central nervous systems. The dose of 50 Gy is higher than the acceptable astronauts’ exposure limits [39], and it was selected in this work to assess the shielding robustness of MDPE nanocomposites. The tests (Figure 2) were conducted at the Crocker Nuclear Laboratory of the University of California (Davis, CA, USA).

The Fourier transform infrared (FTIR) spectra of the nanocomposites before and after proton irradiation were studied using a Thermo-Scientific Nicolet Summit spectrometer equipped with an attenuated total reflection (ATR) accessory (ZnSe crystal). The ATR-FTIR spectra were acquired in the wavenumber range from 400 cm^−1^ to 4000 cm^−1^, at a resolution of 4 cm^−1^ and auto scanning speed of 2 mm/s, keeping the air as a reference. The equation proposed by Zerbi et al. [40] is used to determine the degree of crystallinity (X_c_) from FTIR spectra:

(1) $$X_{c}=100-100\times \frac{1-\left(\frac{I_{a}}{I_{b}}\right)/1.2333}{\left(1+\frac{I_{a}}{I_{b}}\right)}$$

where I_a_ and I_b_ are the intensities of the bands at 730 cm^−1^ and 720 cm^−1^, respectively. The constant 1.2333 corresponds to the relation of the intensities of these bands in the fully crystalline polyethylene spectrum [41].

Thermal analysis was performed using a double-furnace differential scanning calorimeter (DSC 8500, PerkinElmer, Waltham, MA, USA). Samples were sealed in aluminum pans with lids and measured in the temperature range from −45 °C to 150 °C with heating and cooling rates of 10 °C/min under nitrogen flow (20 cc/min). The degree of crystallinity was evaluated from the melting enthalpies (ΔH_m_) determined as the area under the melting peak in the DSC thermograms, using the following equation:

(2) $$X_{c}=100\times \Delta H_{m}/\Delta H_{f}\left(1-w_{f}\right)$$

where ΔH_f_ is the latent heat of fusion of 100% crystalline polyethylene (288 J/g) [42] and w_f_ is the weight fraction of the nanoparticles.

The surface wettability of the nanocomposites was characterized by static contact angles (CA) measurements at the top surface using a DataPhysics OCA15Pro analyzer (DataPhysics Instruments, Filderstadt, Germany). The measurements were performed with the sessile drop method using degassed ultrapure water and diiodomethane as testing liquids. The determination of the contact angle values was performed according to the Young–Laplace fitting method using the DataPhysics SCA20 image analysis software. The values of the surface free energy (SFE) were determined with the Owens–Wendt method [43]:

(3) $$\gamma \left(1+\cos\theta \right)=2\left[{\left(\gamma_{s}^{d}\gamma_{l}^{d}\right)}^{\frac{1}{2}}+{\left(\gamma_{s}^{p}\gamma_{l}^{p}\right)}^{\frac{1}{2}}\right]$$

where γ_s_ is the SFE of the solid that is analyzed, γ_l_ is the SFE of the measuring liquid, the apexes d and p indicate the dispersive and polar components, respectively, and θ is the contact angle between the solid and the testing liquid. The reported values of the CA and SFE are the average of ten measurements.

The morphology of the nanocomposites before and after proton irradiation was investigated using a VEGA II LSH scanning electron microscope (TESCAN, Brno, Czech Republic) with an accelerating voltage of 5 kV and a magnification of 500×. SEM images were acquired before and after proton exposure. The MountainsMap 7 software (Digital Surf, Besançon, France) was used to perform a 3D reconstruction of the specimen surface and the evaluation of surface roughness (R_a_) from images acquired at different tilt angles (0° and 1°) [44]. The R_a_ was averaged over values determined on profiles extracted every 0.1 mm across the reconstructed 3D surface, typically 30 profiles for a surface area of 300 μm × 300 μm.

## 3. Results and Discussion

### 3.1. Electrical Properties

The volumetric electrical conductivity of MDPE/GNP at 2.5 wt%, 5 wt%, 10 wt%, and 15 wt%, of MDPE/MWCNT at 2.5 wt% and 5 wt%, and of MDPE/GNP_3_MWCNT_1_ at 20 wt% is given in Table 1. First, a significant enhancement of the electrical properties was observed, with the loading fraction increasing from 2.5 wt% to 5 wt% for both the MDPE/GNP and MDPE/MWCNT systems. Overall, the MDPE/MWCNT system shows higher electrical conductivities than the MDPE/GNP system at all mass concentrations due to the presence of the MWCNTs with their high aspect ratio. In addition, the difference between the electrical conductivity of the MDPE/GNP at 5 wt%, 10 wt%, and 15 wt% is negligible, meaning that further increasing the nanofiller concentration has a minimal effect. As regards the MDPE/GNP_3_MWCNT_1_ 20 wt% nanocomposite, it shows the highest electrical conductivity among the investigated systems due to the synergistic effect of the GNPs and CNTs nanoparticles: the high aspect ratio of MWCNTs is responsible for the high electrical conductivity and for preventing the restacking of the GNPs, while the GNPs inhibit the aggregation of the MWCNTs, creating an interconnected hybrid architecture [45]. In order to explore the potential applications of carbon-based multifunctional nanocomposites with high electrical conductivity in space radiation environments and the effects of different nanofiller loadings on the radiation sensitivity of these materials, samples (5 cm × 5 cm × 5 cm) of MDPE/GNP at 5 wt%, 10 wt%, and 15 wt%, of MDPE/MWCNT at 5 wt%, and of MDPE/GNP_3_MWCNT_1_ 20 wt% were subjected to proton radiation and their properties were investigated in terms of chemical structure, thermal behavior, wettability, and morphology.

### 3.2. FTIR Analysis

Samples (5 cm × 5 cm × 5 cm) of MDPE/GNP at 5 wt%, 10 wt%, and 15 wt%, of MDPE/MWCNT at 5 wt%, and of MDPE/GNP_3_MWCNT_1_ at 20 wt% were subjected to proton radiation and the chemical changes induced by the exposure were identified by ATR-FTIR, obtaining the spectra (raw data) reported in Figures S1 and S2. The main absorption peaks of polyethylene are given in Table 2, in agreement with the literature [46].

**Figure 3:**
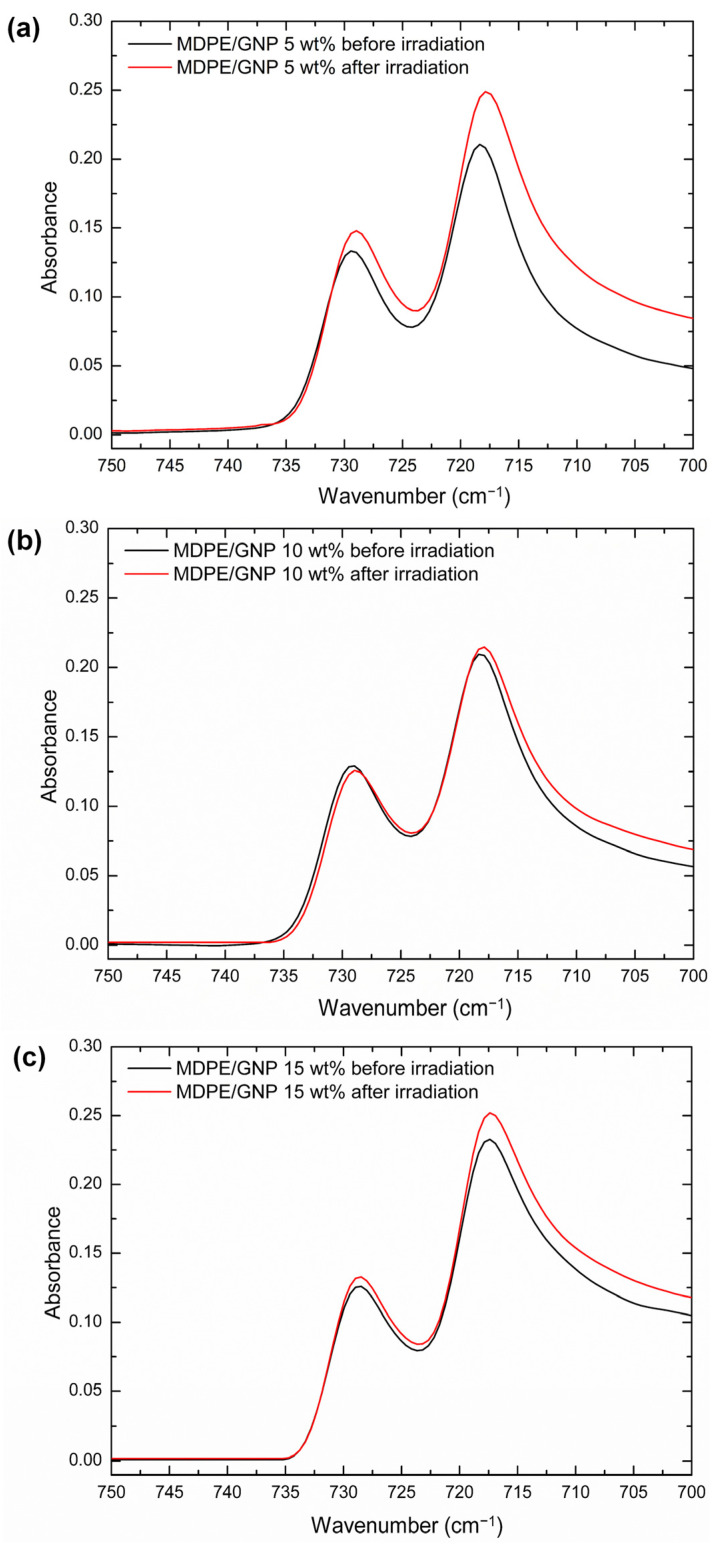
ATR-FTIR spectra of (**a**) MDPE/GNP 5 wt%, (**b**) MDPE/GNP 10 wt%, (**c**) MDPE/GNP 15 wt% nanocomposites before and after proton irradiation in the 750–700 cm^−1^ region.

**Figure 4:**
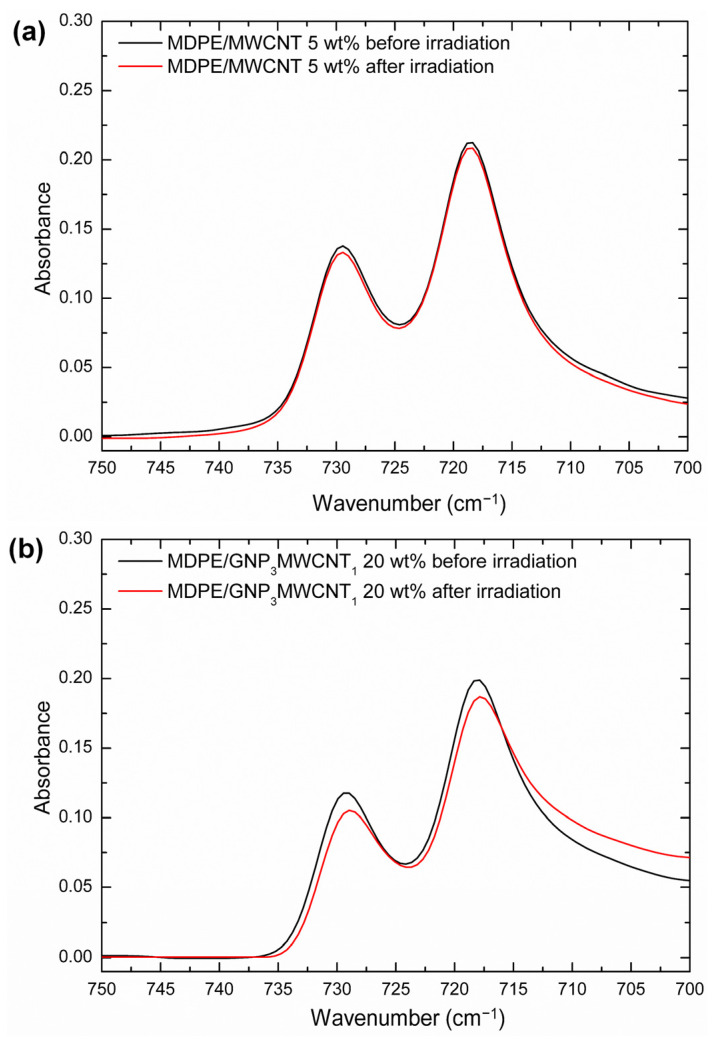
ATR-FTIR spectra of (**a**) MDPE/MWCNT 5 wt% and (**b**) MDPE/GNP_3_MWCNT_1_ nanocomposites before and after proton irradiation in the 750–700 cm^−1^ region.

The addition of GNP and MWCNT nanofillers did not significantly modify the shape of the vibrational spectra of the MDPE matrix. However, two main effects induced by irradiation in atmosphere are observed: oxidative degradation and crystallinity changes. Figure 3 and Figure 4 show ATR-FTIR spectra of the nanocomposites before and after proton irradiation in the 750–700 cm^−1^ region, where the doublet at 718–729 cm^−1^ was analyzed. Table 3 illustrates the X_c_ of the nanocomposites before and after proton irradiation, calculated using the equation proposed by Zerbi et al. [40]. The intensities of the bands at 729 cm^−1^ and 718 cm^−1^ and their ratios are reported in Table S1.

First, a slight decrease in X_c_ is observed as the GNP content increases, which can be attributed to the tendency of GNP to hinder the molecular mobility of the polymer matrix at relatively high concentrations (above 3–5 wt%) [47,48], thus limiting the growth of polyethylene crystallites [48]. The MDPE/MWCNT 5 wt% samples show slightly higher X_c_ values than the MDPE/GNP 5 wt% nanocomposites since the GNP filler imposes more constraints around the polymer chains, inducing a greater fraction of polymer chains to be trapped in the graphene network [49]. All nanocomposites show a decrease in X_c_ after exposure to proton radiation; however, this effect is negligible for the MDPE/MWCNT 5 wt% sample. Furthermore, as the filler content increases, the relative percentage variations in X_c_ (indicated as ΔX_c_/X_c_) decrease, confirming an increasing shielding effect with increasing filler content. Overall, the decrease of X_c_ after irradiation can be explained by branching and cross-linking phenomena, in accordance with the current literature [50,51,52,53]. In the case of irradiation of polymeric materials, macro radicals will be generated both in the amorphous and crystalline phases. These radicals can then react with molecular or atomic oxygen leading to the formation of ketones, aldehydes, alcohols, and carboxylic acids. The selected GNP nanoparticles have nitrogen and oxygen atoms attached to the graphene sheets that are likely cleaved when irradiated [54,55]. The generated free radicals can react with polyethylene, leading to the formation of cross-linked bonds in the side chains of the polymer matrix. In this way, the increase of short-chain branching density decreases the lamellar thickness of the crystal structure, consequently reducing the X_c_ of irradiated samples [53,56].

### 3.3. Thermal Analysis by Differential Scanning Calorimetry

**Figure 5:**
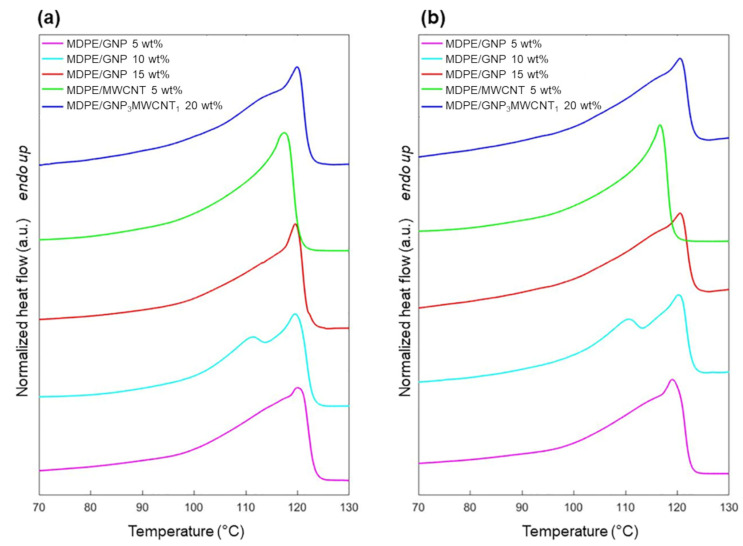
DSC thermograms upon heating for MDPE/GNP 5 wt%, MDPE/GNP 10 wt%, MDPE/GNP 15 wt%, MDPE/MWCNT 5 wt% and MDPE/GNP_3_MWCNT_1_ 20 wt% nanocomposites (**a**) before and (**b**) after proton irradiation. Heating rate: 10 °C/min. Heat flow is normalized by the sample weight. Data are offset for clarity.

**Figure 6:**
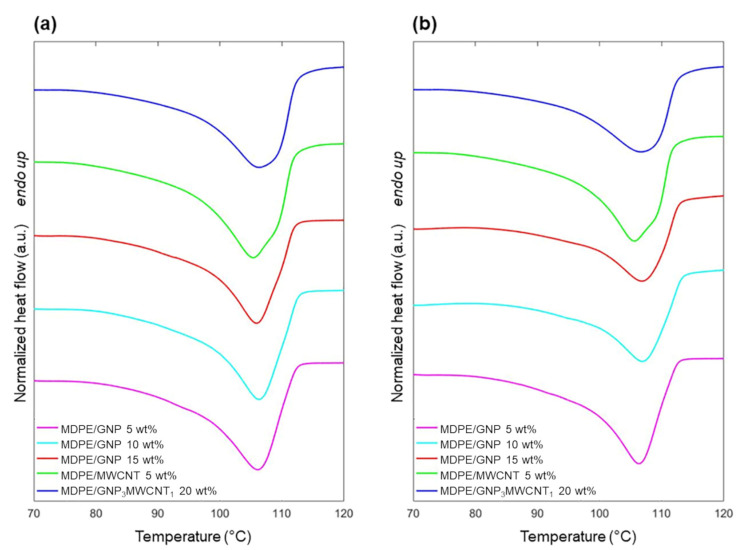
DSC thermograms upon cooling for MDPE/GNP 5 wt%, MDPE/GNP 10 wt%, MDPE/GNP 15 wt%, MDPE/MWCNT 5 wt% and MDPE/GNP_3_MWCNT_1_ 20 wt% nanocomposites (**a**) before and (**b**) after proton irradiation. Heating rate: 10 °C/min. Heat flow is normalized by the sample weight. Data are offset for clarity.

The thermal behavior of the nanocomposites was analyzed by DSC before and after proton irradiation. Thermograms of heating and cooling of the samples are reported in Figure 5 and Figure 6, respectively. Results from thermal analysis are summarized in Table 4 and Table 5. As the samples were irradiated in a solid state, the first heating cycle was used to investigate changes in crystallinity induced by radiation, and the results were compared with those obtained by FTIR.

As shown in Table 4, only small differences in the values of the melting (T_m_) and crystallization (T_c_) temperatures are observed among the different nanocomposites and after the radiation process. Results regarding the degree of crystallinity calculated by DSC (Table 5) confirm the same trend showed by ATR-FTIR analysis. In fact, also, in this case, comparing the X_c_ values before and after proton exposure, a decrease in crystallinity caused by the irradiation can be observed, and this is more evident in the presence of the GNP filler. Further, the presence of a shoulder in the nanocomposites containing GNP was observed (Figure 5), and it can be ascribed to the melting of imperfect crystals. This phenomenon can be related to the more constraints imposed by the GNP filler on the polymer matrix [49], favoring the formation of imperfect crystalline lamellae. For the samples containing GNPs, the decrease of ΔH_c_ upon irradiation confirms that the formation of cross-links and chain branches hinders the polymer chain's mobility and chain reorganization during the crystallization process, leading to the formation of imperfect and thinner lamellae [57]. As anticipated in the ATR-FTIR analysis, the values of X_c_ and ΔH_c_ of the MDPE/MWCNT 5 wt% nanocomposite are less affected by proton irradiation. Although the trend in the degree of crystallinity is the same, X_c_ values determined by FTIR are higher for all samples. This has already been reported in the literature and can be mainly ascribed to some limitations of the DSC technique. In fact, the crystallinity of the polymer is temperature dependent, and the estimated crystallinity determined by DSC at the melting temperature will differ from the value at ambient temperature [48,58]. Moreover, the differential nature and overlap of multiple thermal events (chain relaxation, melting of different crystal forms) can affect the values of X_c_ determined by DSC. Nevertheless, the DSC technique allows for detecting bulk features, whereas ATR-FTIR mainly reveals the surface characteristics of the material. Overall, these two techniques should be considered complementary to better understand the behavior of the nanocomposites. In fact, it is possible that irradiation induced chain-branching and cross-linking on top of the irradiated surface, while chain scission is predominant in the bulk of the sample.

### 3.4. Contact Angle Measurements

The wetting behavior before and after proton irradiation was investigated by static contact angle measurements in sessile drop configuration. The water contact angle (WCA) and SFE values are given in Table 6, showing an alteration of the WCA and SFE of irradiated samples compared with the non-irradiated ones. The analysis revealed the hydrophobicity of the non-irradiated nanocomposites, which are characterized by WCA values above 105°. The WCA strongly increases with nanofiller content, reaching 115.7° ± 2.5° for the hybrid nanocomposite. After proton exposure, the WCA of all nanocomposites decreased, with the largest variation in surface wettability observed in the MDPE/MWCNT 5 wt% nanocomposite (11.1%). The surface hydrophobicity of the PE/GNP nanocomposites was reduced in proportion to the GNP content, with the smallest WCA decrease (3.2%) for the MDPE/GNP 5 wt% surface and the largest (8.4%) for the MDPE/GNP 15 wt% surface. It is known that the WCA of a polymer surface depends on chemical functional groups and asperities: it decreases with increasing surface energy and surface smoothness [59]. Hence, to further investigate the decrease of the WCA values occurring upon irradiation, a surface-free energy analysis following the Owens–Wendt method [43] was carried out with two different testing liquids (water and diiodomethane). As shown in Table 6, the SFE decreases at increasing nanofiller content in non-irradiated samples. The dispersive component (γ^d^), which is due to the dispersive interactions among non-polar molecules, is predominant over the polar one (γ^p^) for all investigated samples. After proton exposure, the SFE and its dispersive component show a marked increase in the nanocomposite samples. On the contrary, the polar component decreases in GNP-loaded nanocomposites, and increases in the MDPE/MWCNT 5 wt% nanocomposite. The decrease of surface hydrophobicity upon irradiation is in agreement with reports in the literature [21,22,60]. It can be ascribed to an oxidation phenomenon in the MDPE/MWCNT 5 wt% nanocomposite (as revealed by the increase of γ^p^) and to the formation of nonpolar chain branches in GNP-loaded nanocomposites (as revealed by the increase of γ^d^). In fact, it was demonstrated that the oxygen content in proton-irradiated MWCNTs is higher than in non-irradiated ones [61]. This behavior has not been observed for the MDPE/GNP_3_MWCNT_1_ 20 wt% samples, and this can be explained by the predominant presence of GNP (GNP/MWCNT ratio of 3:1), which may plausibly mitigate this phenomenon. In addition, the morphological analysis of the nanocomposite surface reported below shows the improved surface smoothness of irradiated samples, which also contributes to the decrease of the WCA [59].

### 3.5. Morphological Analysis

**Figure 7:**
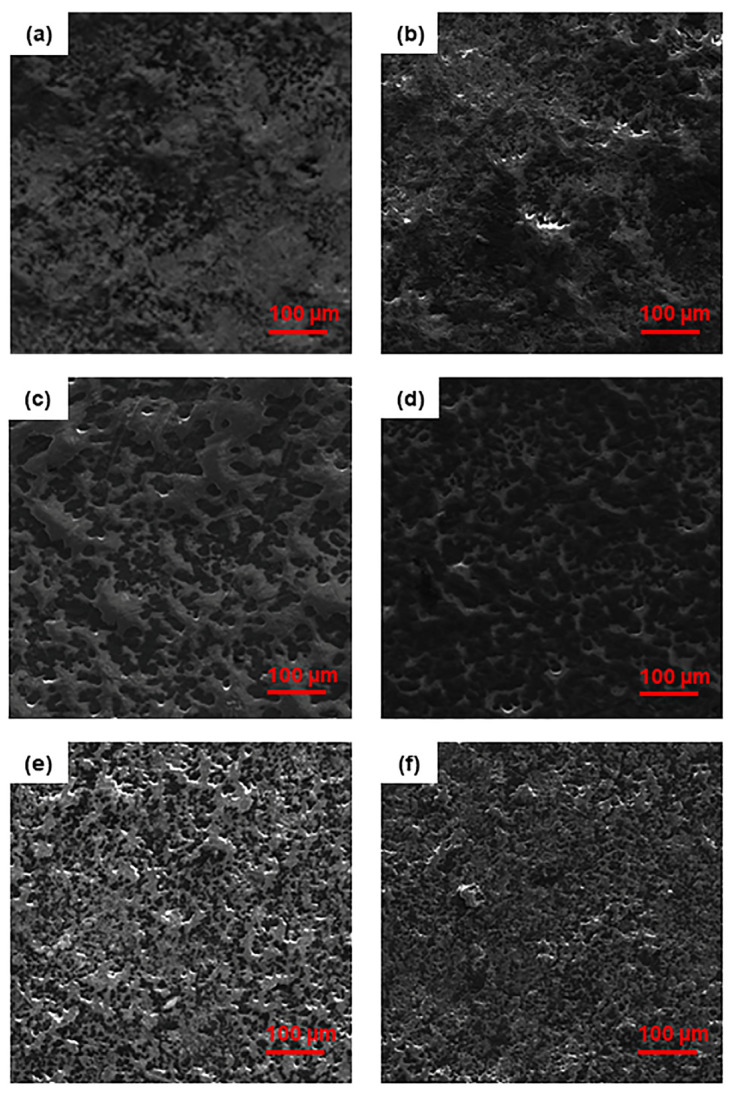
SEM images of non-irradiated (**left**) and irradiated (**right**) samples of (**a**,**b**) MDPE/GNP 5 wt%, (**c**,**d**) MDPE/GNP 10 wt%, (**e**,**f**) MDPE/GNP 15 wt% nanocomposites.

**Figure 8:**
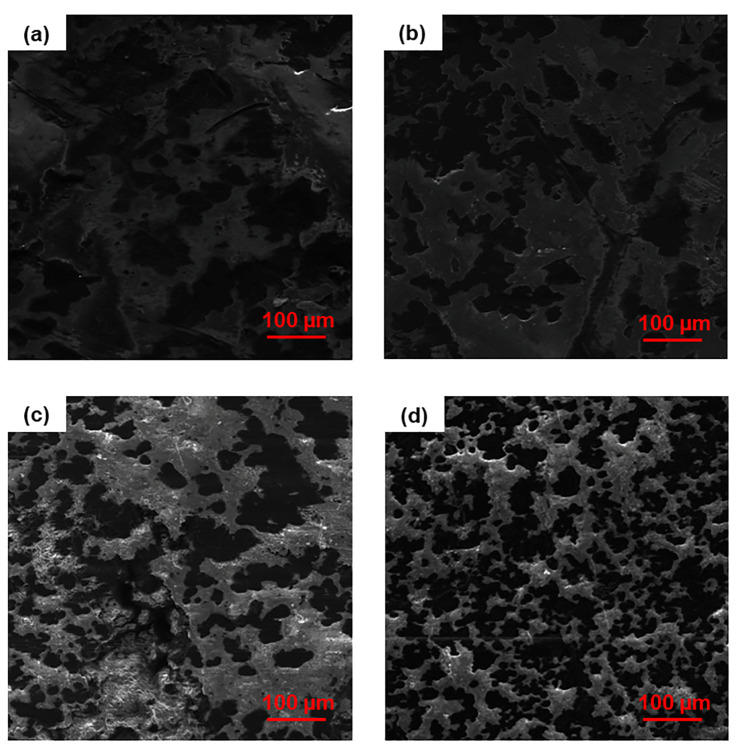
SEM images of non-irradiated (**left**) and irradiated (**right**) samples of (**a**,**b**) MDPE/MWCNT 5 wt% and (**c**,**d**) MDPE/GNP_3_MWCNT_1_ 20 wt% nanocomposites.

The surface morphology of the nanocomposites before and after proton irradiation was investigated by SEM (Figure 7 and Figure 8). The images reveal the erosive effect of radiation that is more evident at high nanofiller loadings, with the surfaces of the nanocomposites appearing smoother after exposure.

**Figure 9:**
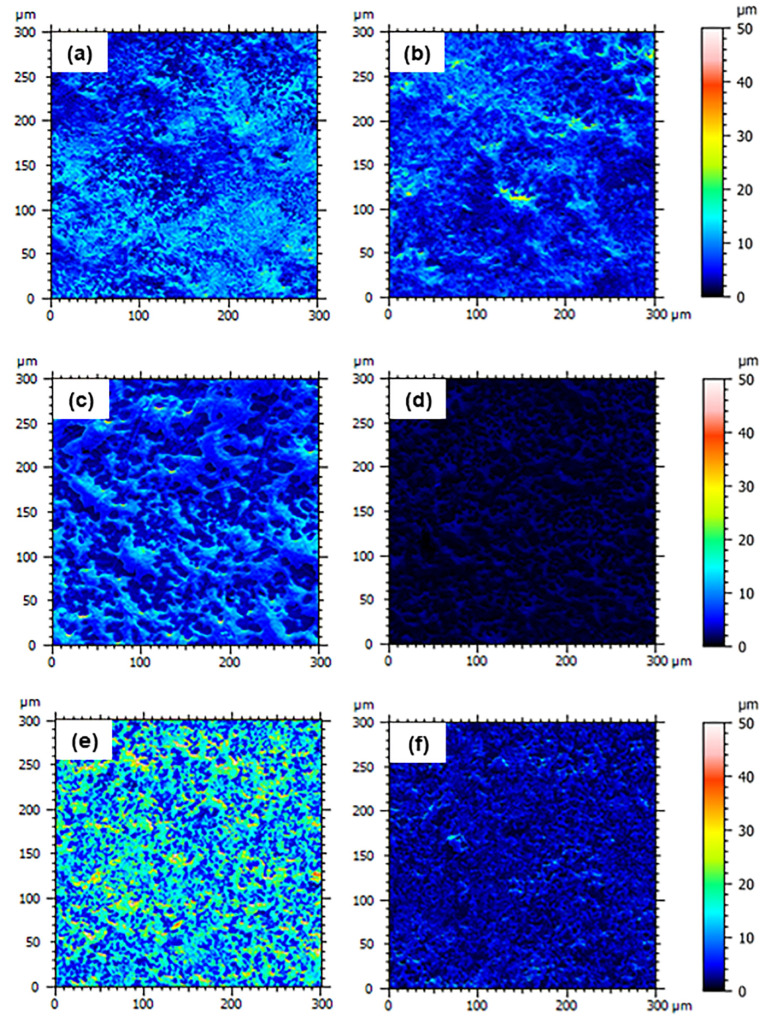
3D surface reconstruction through SEM image processing of non-irradiated (**left**) and irradiated (**right**) samples of (**a**,**b**) MDPE/GNP 5 wt%, (**c**,**d**) MDPE/GNP 10 wt%, (**e**,**f**) MDPE/GNP 15 wt% nanocomposites.

**Figure 10:**
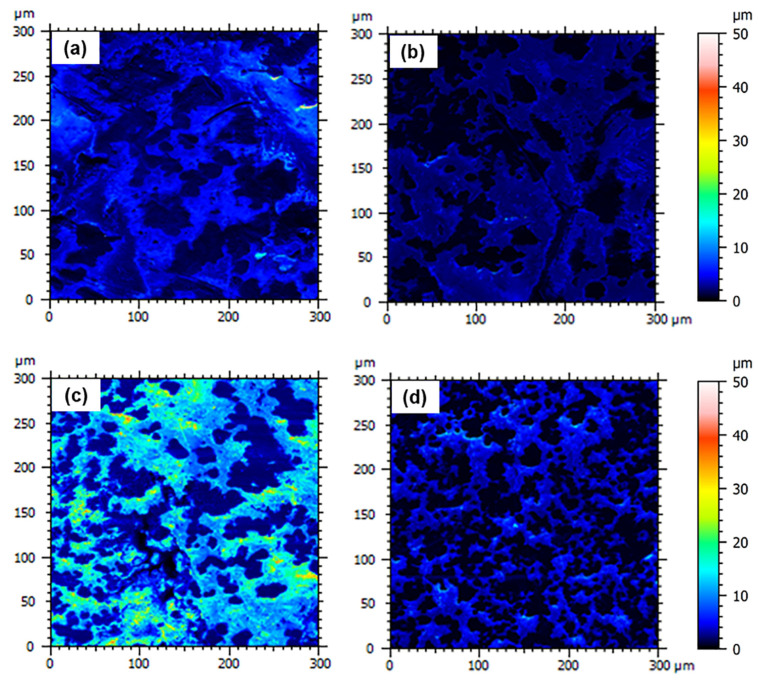
3D surface reconstruction through SEM image processing of non-irradiated (**left**) and irradiated (**right**) samples of (**a**,**b**) MDPE/MWCNT 5 wt% and (**c**,**d**) MDPE/GNP_3_MWCNT_1_ 20 wt% nanocomposites.

Due to the formation of entanglements at high nanofiller contents, we find an increasing number of surface asperities with increasing filler concentrations. In particular, we observe a high number of small peaks on the surface of MDPE/GNP specimens. By contrast, the surface morphology of the MDPE/MWCNT 5 wt% and MDPE/GNP_3_MWCNT_1_ 20 wt% samples is characterized by the presence of large peaks. The reason is attributed to the large aggregates formed due to the weak interaction of the pristine MWCNTs with the MDPE matrix and to the van der Waals forces acting among the MWCNT nanoparticles. A 3D reconstruction of the surface profiles was performed to quantify the effect of the radiation exposure at the top surface of the nanocomposite samples. 3D images were obtained with the Mountains Map software, starting from two SEM images acquired at two different tilt angles (0° and 1°). Results of the 3D surface reconstruction are reported in Figure 9 and Figure 10. A decrease in the height of surface peaks in irradiated nanocomposites is clearly visible on the color-scaled SEM images, which corresponds to an increased surface smoothness.

Table 7 presents the R_a_ values of the nanocomposites before and after proton irradiation. Despite the different nanofiller concentrations, the MDPE/GNP_3_MWCNT_1_ 20 wt% and MDPE/GNP 15 wt% non-irradiated nanocomposites show similar values of the R_a_. This result is explained by the presence of both GNP and MWCNT nanofillers in the hybrid system, where the graphene nanoplatelets prevent the aggregation of nanotubes by physically hindering the process due to their large surface area [45]. All nanocomposites show a significant decrease of R_a_ upon exposure to proton radiation, with the largest variation observed in the MDPE/GNP 15 wt% (76.5%). This result confirms the cleavage effect of irradiation on the edges of the GNPs and subsequent surface erosion.

## 4. Conclusions

In this work, the sensitivity to proton radiation of nanocomposites made of medium-density polyethylene (MDPE) loaded with different amounts of GNP and MWCNT nanofillers was investigated. Different techniques were used to study the nanocomposite’s response in terms of chemical structure, thermal behavior, wettability, and morphology. Results regarding the degree of crystallinity evaluated by FTIR and DSC unveiled a higher decrease of crystallinity after proton irradiation in the nanocomposites filled with GNP due to the branching and cross-linking mechanisms induced by the radiation. The MDPE/MWCNT 5 wt% nanocomposite showed the highest degree of crystallinity, with unnoteworthy changes after irradiation. The DSC analysis showed the thermal stability of the investigated nanocomposites in response to radiation, with T_m_ and T_c_ that remained unaltered. Despite a decrease in the WCA values of all nanocomposites after proton exposure, all samples maintained a hydrophobic surface and, therefore, a low tendency to adsorb water vapor from the environment. Lastly, a 3D reconstruction of the surface profiles was performed to quantify the effect of the radiation exposure at the top surface of the nanocomposite samples, revealing an increased surface smoothness after irradiation. Nevertheless, this effect is less marked in the MDPE/GNP 5 wt% and MDPE/MWCNT 5 wt% nanocomposites.

Overall, the results of this work show that the MDPE/MWCNT 5 wt% material is the best MDPE/nanocarbon system for use in radiation shielding applications due to the negligible changes observed in the physico-chemical properties after proton irradiation.

## Figures and Tables

**Table 1:** Electrical conductivity of MDPE/GNP and MDPE/MWCNT samples at different filler concentrations, and MDPE/GNP_3_MWCNT_1_ 20 wt% nanocomposite.

Sample	σ_v_ (S/mm)
MDPE/GNP 2.5 wt%	1.81 × 10^−14^ ± 5.1 × 10^−15^
MDPE/GNP 5 wt%	2.10 × 10^−13^ ± 1.4 × 10^−14^
MDPE/GNP 10 wt%	1.53 × 10^−12^ ± 3.9 × 10^−14^
MDPE/GNP 15 wt%	7.63 × 10^−12^ ± 8.0 × 10^−14^
MDPE/MWCNT 2.5 wt%	6.93 × 10^−9^ ± 4.6 × 10^−10^
MDPE/MWCNT 5 wt%	2.26 × 10^−7^ ± 1.1 × 10^−8^
MDPE/GNP_3_MWCNT_1_ 20 wt%	2.86 × 10^−7^ ± 1.0 × 10^−8^

**Table 2:** Main absorption bands of polyethylene in the IR region and their assignments.

Band (cm^−1^)	Assignment
2914	CH_2_ asymmetric deformation
2847	CH_2_ symmetric deformation
1472, 1462	Bending deformation
1376	CH_2_ symmetric deformation
729, 718	Rocking deformation

**Table 3:** Degree of crystallinity (X_c_) and relative percentage variation (ΔX_c_/X_c_) calculated from FTIR spectra of MDPE/GNP and MDPE/MWCNT nanocomposites at different filler concentrations and MDPE/GNP_3_MWCNT_1_ 20 wt% sample before and after proton irradiation. Standard deviation of data is below 2%.

Sample	X_c_ (%) (by FTIR)		
	Non-Irradiated	Irradiated	ΔX_c_/X_c_ (%)
MDPE/GNP 5 wt%	70.0	67.5	−3.6
MDPE/GNP 10 wt%	69.1	66.9	−3.2
MDPE/GNP 15 wt%	63.6	62.6	−1.6
MDPE/MWCNT 5 wt%	71.4	70.6	−1.1
MDPE/GNP_3_MWCNT_1_ 20 wt%	67.4	65.1	−3.4

**Table 4:** DSC results for MDPE/GNP and MDPE/MWCNT samples at different filler concentrations, and for the MDPE/GNP_3_MWCNT_1_ 20 wt% nanocomposite before and after proton irradiation. Standard deviation of data are below 2%.

**Non-Irradiated**	**T_m_ (°C)**	**ΔH_m_ (J/g)**	**T_c_ (°C)**	**ΔH_c_ (J/g)**
MDPE/GNP 5 wt%	120.0	147.3	106.1	125.8
MDPE/GNP 10 wt%	119.5	137.2	106.3	121.4
MDPE/GNP 15 wt%	119.6	120.9	106.0	115.3
MDPE/MWCNT 5 wt%	117.5	152.4	105.4	141.0
MDPE/GNP_3_MWCNT_1_ 20 wt%	120.0	127.9	106.4	121.6
**Irradiated**	**T_m_ (°C)**	**ΔH_m_ (J/g)**	**T_c_ (°C)**	**ΔH_c_ (J/g)**
MDPE/GNP 5 wt%	119.0	137.1	106.4	119.8
MDPE/GNP 10 wt%	120.2	128.7	107.0	110.2
MDPE/GNP 15 wt%	120.1	114.7	107.0	109.0
MDPE/MWCNT 5 wt%	116.6	148.5	105.6	136.7
MDPE/GNP_3_MWCNT_1_ 20 wt%	120.5	123.9	106.8	113.5

**Table 5:** Degree of crystallinity (X_c_) and relative percentage variation (ΔX_c_/X_c_) calculated by DSC of MDPE/GNP and MDPE/MWCNT nanocomposites at different filler concentrations and MDPE/GNP_3_MWCNT_1_ 20 wt% sample before and after proton irradiation. Standard deviation of data is below 2%.

Sample	X_c_ (%) (by DSC)		
	Non-Irradiated	Irradiated	ΔX_c_ /X_c_ (%)
MDPE/GNP 5 wt%	53.8	50.1	−6.9
MDPE/GNP 10 wt%	52.9	49.7	−6.2
MDPE/GNP 15 wt%	49.4	46.9	−5.2
MDPE/MWCNT 5 wt%	55.7	54.3	−2.6
MDPE/GNP_3_MWCNT_1_ 20 wt%	55.5	53.8	−3.1

**Table 6:** Water contact angles (WCA) and surface free energies (SFE) with dispersive (γ^d^) and polar (γ^p^) components of MDPE/GNP 5 wt%, MDPE/GNP 10 wt%, MDPE/GNP 15 wt%, PE/MWCNT 5 wt% and MDPE/GNP_3_MWCNT_1_ 20 wt% before and after proton irradiation.

**Non-Irradiated**	**WCA (°)**	**SFE (mJ/m^2^)**	**γ^p^**	**γ^d^**
MDPE/GNP 5 wt%	110.1 ± 1.8	36.25	0.19	36.06
MDPE/GNP 10 wt%	112.5 ± 1.1	31.92	0.16	31.76
MDPE/GNP 15 wt%	113.1 ± 1.7	31.33	0.17	31.16
MDPE/MWCNT 5 wt%	105.4 ± 1.8	31.03	0.05	30.98
MDPE/GNP_3_MWCNT_1_ 20 wt%	115.7 ± 2.5	29.02	0.24	28.78
**Irradiated**	**WCA (°)**	**SFE (mJ/m^2^)**	**γ^p^**	**γ^d^**
MDPE/GNP 5 wt%	106.6 ± 1.6	36.44	0.03	36.42
MDPE/GNP 10 wt%	104.3 ± 2.0	32.97	0.05	32.92
MDPE/GNP 15 wt%	103.6 ± 5.3	35.81	0.02	35.80
MDPE/MWCNT 5 wt%	93.7 ± 1.1	36.43	0.98	35.45
MDPE/GNP_3_MWCNT_1_ 20 wt%	103.0 ± 2.8	34.17	0.07	34.10

**Table 7:** Surface roughness and relative percentage variation (ΔR_a_/R_a_) of MDPE/GNP 5 wt%, MDPE/GNP 10 wt%, MDPE/GNP 15 wt%, MDPE/MWCNT 5 wt% and MDPE/GNP_3_MWCNT_1_ 20 wt% nanocomposites before and after proton irradiation.

Sample	R_a_ (µm)		
	Non-Irradiated	Irradiated	ΔR_a_/R_a_ (%)
MDPE/GNP 5 wt%	3.6 ± 0.5	2.6 ± 0.4	−27.8
MDPE/GNP 10 wt%	6.7 ± 1.6	2.2 ± 0.3	−67.2
MDPE/GNP 15 wt%	5.1 ± 0.6	1.2 ± 0.2	−76.5
MDPE/MWCNT 5 wt%	3.6 ± 0.6	2.4 ± 0.3	−33.3
MDPE/GNP_3_MWCNT_1_ 20 wt%	5.1 ± 0.8	2.1 ± 0.3	−58.8

## Data Availability

Data are contained within the article or Supplementary Material.

## Supplementary Materials

The following supporting information can be downloaded at: https://www.mdpi.com/article/10.3390/nano13071288/s1, Figure S1: ATR-FTIR spectra (raw data) of (**a**) MDPE /GNP 5 wt%, (**b**) MDPE /GNP 10 wt% and (**c**) MDPE/GNP 15 wt% nanocomposites before and after proton irradiation. Data are offset for clarity; Figure S2: ATR-FTIR spectra (raw data) of (**a**) MDPE/MWCNT 5 wt% and (**b**) MDPE/GNP_3_MWCNT_1_ 20 wt% nanocomposites before and after proton irradiation. Data are offset for clarity; Table S1: ATR-FTIR data of non-irradiated and irradiated nanocomposites: intensity of the band at 729 cm^−1^ (I_a_) and 718 cm^−1^ (I_b_) and their ratio (I_a_/I_b_).
