# Supplementary material for: Impact of Proton Irradiation on Medium Density Polyethylene/Carbon Nanocomposites for Space Shielding Applications
Source: Nanomaterials (Basel). 2023 Apr 6;13(7):1288. doi: 10.3390/nano13071288 (PMC10097310; doi:10.3390/nano13071288)
Supplement: Supplementary file 1 [file nanomaterials-13-01288-s001.zip › nanomaterials-2276139-supplementary.pdf]

Supplementary Materials

Impact of Proton Irradiation on Medium Density Polyethylene/Carbon Nanocomposites for Space Shielding Applications

Federica Zaccardi [1,†], Elisa Toto [2,†], Shreya Rastogi [3], Valeria La Saponara [3], Maria Gabriella Santonicola [2] and Susanna Laurenzi [1,*]

1 Department of Astronautical Electrical and Energy Engineering, Sapienza University of Rome, Via Salaria 851-881, 00138 Rome, Italy; federica.zaccardi@uniroma1.it
2 Department of Chemical Engineering Materials Environment, Sapienza University of Rome, Via del Castro Laurenziano 7, 00161 Rome, Italy; elisa.toto@uniroma1.it (E.T.); mariagabriella.santonicola@uniroma1.it (M.G.S.)
3 Department of Mechanical and Aerospace Engineering, University of California Davis, One Shields Ave, Davis, CA 95616, USA; vlasaponara@ucdavis.edu (V.L.S.)
* Correspondence: susanna.laurenzi@uniroma1.it
† These authors contributed equally.

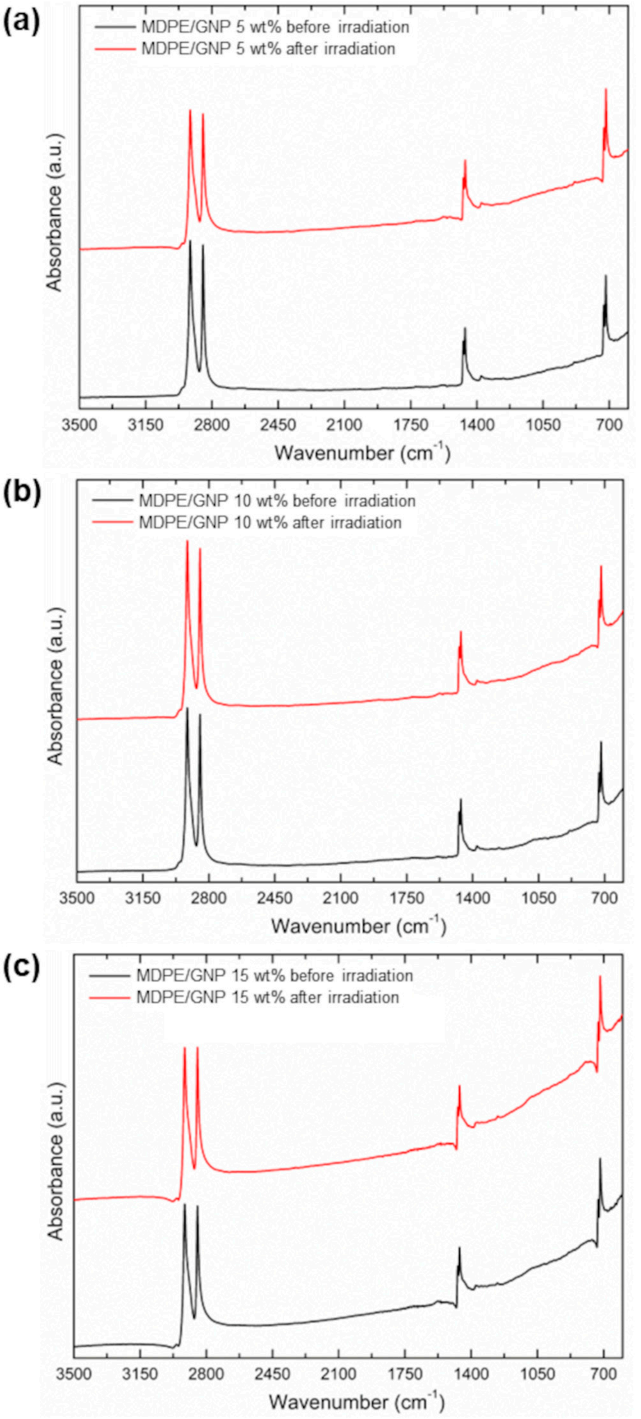

Figure S1. ATR-FTIR spectra (raw data) of (**a**) MDPE/GNP 5 wt%, (**b**) MDPE /GNP 10 wt% and (**c**) MDPE/GNP 15 wt% nanocomposites before and after proton irradiation. Data are offset for clarity.

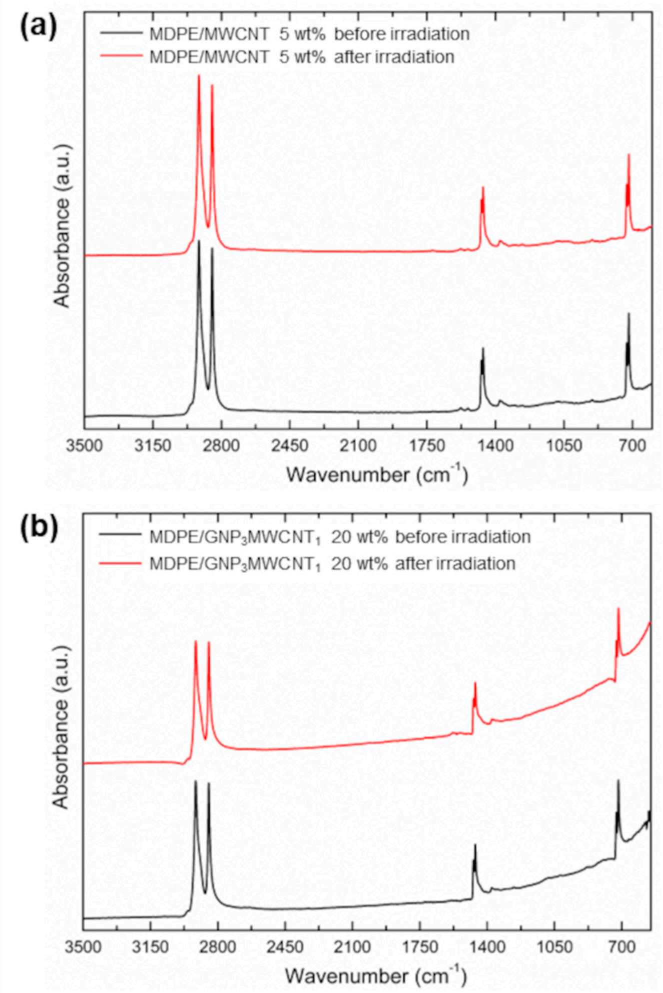

Figure S2. ATR-FTIR spectra (raw data) of (**a**) MDPE/MWCNT 5 wt% and (**b**) MDPE/GNP_3MWCNT_1 20 wt% nanocomposites before and after proton irradiation. Data are offset for clarity.

Table S1. ATR-FTIR data of non-irradiated and irradiated nanocomposites: intensities of the 729 cm^{-1} (I_a) and 718 cm^{-1} (I_b) peaks and their ratio (I_a/I_b).

**Non-irradiated**	**I_a**	**I_b**	**I_a/I_b**
MDPE/GNP 5 wt%	0.133	0.211	0.630
MDPE/GNP 10 wt%	0.129	0.209	0.617
MDPE/GNP 15 wt%	0.126	0.233	0.541
MDPE/MWCNT 5 wt%	0.138	0.212	0.651
MDPE/GNP_3MWCNT_1 20 wt%	0.118	0.199	0.593
**Irradiated**	**I_a**	**I_b**	**I_a/I_b**
MDPE/GNP 5 wt%	0.148	0.249	0.594
MDPE/GNP 10 wt%	0.126	0.215	0.586
MDPE/GNP 15 wt%	0.133	0.252	0.528
MDPE/MWCNT 5 wt%	0.133	0.208	0.639
MDPE/GNP_3MWCNT_1 20 wt%	0.105	0.187	0.561

I_a: intensity of the band at 729 cm^{-1}; I_b: intensity of the band at 718 cm^{-1}
